# A novel porcine reproductive and respiratory syndrome virus vector system that stably expresses enhanced green fluorescent protein as a separate transcription unit

**DOI:** 10.1186/1297-9716-44-104

**Published:** 2013-10-31

**Authors:** Chengbao Wang, Baicheng Huang, Ning Kong, Qiongyi Li, Yuping Ma, Zhijun Li, Jiming Gao, Chong Zhang, Xiangpeng Wang, Chao Liang, Lu Dang, Shuqi Xiao, Yang Mu, Qin Zhao, Yani Sun, Fernando Almazan, Luis Enjuanes, En-Min Zhou

**Affiliations:** 1 Department of Preventive Veterinary Medicine, College of Veterinary Medicine, Northwest A&F University, No. 22 Xinong Road, Yangling, Shaanxi 712100, China; 2 Experimental Station of Veterinary Pharmacology and Veterinary Biotechnology, Ministry of Agriculture of the People’s Republic of China, No. 22 Xinong Road, Yangling, Shaanxi 712100, China; 3 Department of Molecular and Cell Biology, C/Darwin no. 3, Campus de Cantoblanco, Spanish National Centre for Biotechnology (CNB-CSIC), 28049, Madrid, Spain

## Abstract

Here we report the rescue of a recombinant porcine reproductive and respiratory syndrome virus (PRRSV) carrying an enhanced green fluorescent protein (EGFP) reporter gene as a separate transcription unit. A copy of the transcription regulatory sequence for ORF6 (TRS6) was inserted between the N protein and 3′-UTR to drive the transcription of the EGFP gene and yield a general purpose expression vector. Successful recovery of PRRSV was obtained using an RNA polymerase II promoter to drive transcription of the full-length virus genome, which was assembled in a bacterial artificial chromosome (BAC). The recombinant virus showed growth replication characteristics similar to those of the wild-type virus in the infected cells. In addition, the recombinant virus stably expressed EGFP for at least 10 passages. EGFP expression was detected at approximately 10 h post infection by live-cell imaging to follow the virus spread in real time and the infection of neighbouring cells occurred predominantly through cell-to-cell-contact. Finally, the recombinant virus generated was found to be an excellent tool for neutralising antibodies and antiviral compound screening. The newly established reverse genetics system for PRRSV could be a useful tool not only to monitor virus spread and screen for neutralising antibodies and antiviral compounds, but also for fundamental research on the biology of the virus.

## Introduction

Porcine reproductive and respiratory syndrome (PRRS), characterized by respiratory diseases in nursery pigs and reproductive failure in sows [1,2], has become one of the most economically important infectious diseases in the global swine industry [3]. PRRS virus (PRRSV), the causative agent of PRRS, is a member of a group of enveloped RNA viruses from the genus Arterivius of the family *Arteriviridae* within the order *Nidovirales*. PRRSV genome is a linear, non-segmented, single stranded, positive-sense RNA with 15 kb in size [4], capped at the 5′ end and polyadenylated at the 3′ terminus. The PRRSV genome consists of at least six genes, encoding the structural proteins GP2 or GP2a, GP3, GP4, GP5, M, and N, which are in the order of 5′-ORF1-GP2-GP3-GP4-GP5-M-N-3′ on the genome [5,6]. Two distinct genotypes of PRRSV, the European genotype (type 1) and North American genotype (type 2) [2,7-10], share only 60% nucleotide identity and are represented by the North American prototype VR-2332 and the European prototype Lelystad virus (LV) [11]. In 2006, the highly pathogenic PRRS (HP-PRRS), characterized by high fever, high morbidity and mortality in pigs of all ages, occurred in China and resulted in the culling of an estimated 20 million pigs in 2006–2007 [12]. The causative agent of HP-PRRS is HP-PRRSV and was more virulent than the classical PRRSV [13]. Genomic analysis showed that nearly all of emerging HP-PRRSV isolates share a unique discontinuous deletion of 30 amino acids (aa) in non-structural protein 2 (Nsp2). However, a number of newly isolated PRRSV variants with different deletions or recombinants was reported in China and the HP-PRRSV variant continues prevailing and accelerating evolution in China [14-16].

Over the past decade, reverse genetics systems developed for the study of many of the positive-stranded RNA viruses can generate recombinant mutant viruses to study different aspects of virus biology. In particular, recombinant viruses expressing reporter proteins have been instrumental in tracking viral infection and spread in vivo, as well as in the development of novel antiviral drugs [17-22]. In PRRSV, rescue systems to recover infectious viruses from full length complementary DNA (cDNA) clones have been established for type 1 and type 2 PRRSV to understand the viral life cycle, determine the function of viral protein, and study mutant virus pathogenesis [23-34]. Green fluorescent protein (GFP) and its variant enhanced GFP (EGFP) inserted into a number of viral genomes have become the widely used reporter genes to study viral entry mechanisms, observe the transport of viral nucleic acids in and out of the nucleus region, track the assembly and transport of newly synthesized viral components, and visualize the cell-to-cell spread of newly synthesized viral particles [35-40].

Several studies have indicated that the presence of overlapping genes in arteritis virus genomes hampers mutational analysis of the N- and C-termini of the structural proteins and also makes it difficult to insert heterologous genes into the viral genome [22,41,42]. The first recombinant HA-tagged PRRSV lost foreign HA epitope fused with the ORF7 from the second passage [43]. The rescued PRRSV with the green fluorescent gene fused with the Nsp2 protein, lost the green fluorescent after 7 serial passages due to the deletion of N-terminal amino acids (1 to 159aa) of GFP [44] or the accumulation of point mutations [45]. In addition, apart from the fusion of the green fluorescent gene with Nsp2, a recombinant PRRSV expressing GFP gene proved the stability of the foreign GFP gene even after 37 serial passages. This was done by using the transcription regulatory sequence of gene 2 (TRS2) to drive transcription of the GFP gene and inserting an extra synthetic TRS6 to drive transcription of the ORF 2a and 2b [22].

In this study, we developed an HP-PRRSV infectious clone using a bacterial artificial chromosome (BAC). This BAC expressed the EGFP gene inserted between the N gene and the 3′UTR region of the viral genome with a copy of TRS6 to drive transcription of the EGFP to create a marker recombinant PPRSV with limited modification of the genome. This recombinant virus stably expresses EGFP as a separate transcription unit and was found to be a useful tool to monitor virus spread, screen for neutralising antibodies and antiviral compounds.

## Materials and methods

### Cell lines, viruses and drugs

HP-PRRSV/SD16 used in this study was isolated from an intensive pig farm with an atypical PRRS outbreak in Shandong province of China in 2012 [16]. The complete cDNA of the HP-PRRSV/SD16 genome was fully sequenced and confirmed [GenBank: JX087437]. Marc-145 cells were cultured in Dulbecco’s modified Eagle’s medium (DMEM) supplemented with 10% fetal bovine serum at 37 °C in a 5% CO_2_ incubator. The cyclophilin inhibitors CsA (Bioaustralis) were dissolved in dimethyl sulfoxide (DMSO). CsA stock at 50 mg/mL was stored at -20 °C.

### Plasmid construction

**Figure 1:**
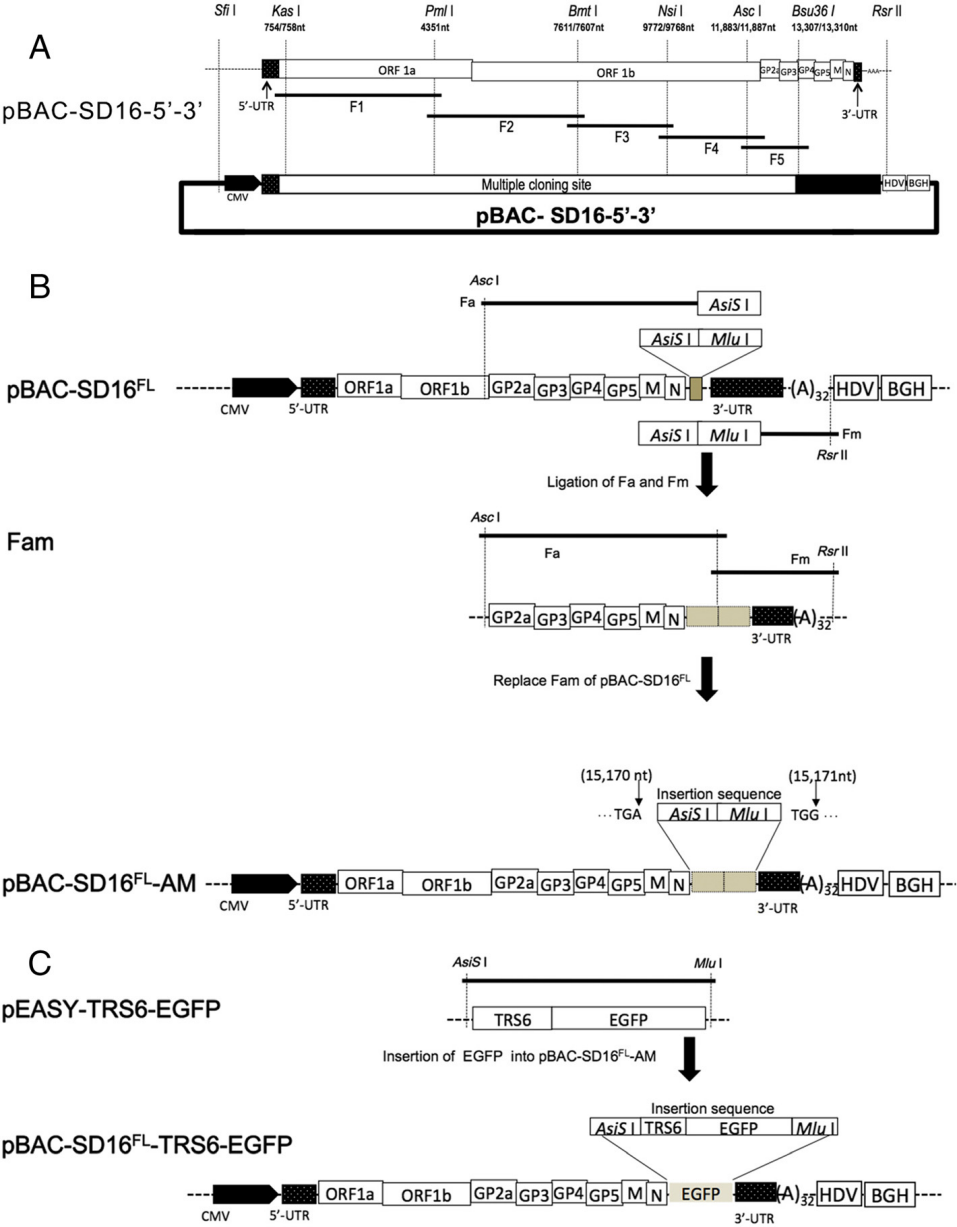
**Construction of plasmids for PRRSV rescue. A**. The cDNA fragments F1, F2, F3, F4, and F5 were reversely transcribed and amplified from HP-PRRSV/SD16 genomic RNA. The CMV promoter was introduced to the 5′ end of HP-PRRSV/SD16 and the hepatitis delta virus ribozyme (HDV) and the bovine growth hormone termination and polyadenylation sequences (BGH) were included at the 3′ end of HP-PRRSV/SD16. All fragments were subcloned stepwise into the pBAC-SD16-5′-3′ vector to produce plasmid pBAC-SD16^FL^. **B**. DNA fragments Fa (from the genome position of 11,883 nt to the stop codon of M with *AsiS* I and *Mlu* I sites introduced at the 3′ end) and Fm (from start sequence of 3-UTR to the pBAC-SD16-5′-3′ vector with *AsiS* I and *Mlu* I sites introduced at the 5′ end) were PCR-amplified from pBAC-SD16^FL^ and ligated together to form fragment Fam. The corresponding fragment of pBAC-SD16^FL^ was replaced with Fam to construct plasmid pBAC-SD16^FL^-AM, which contains *AsiS* I and *Mlu* I sites between nt 15 170 and 15 171 of the HP-PRRSV/SD16 genome cDNA sequence. **C**. The EGFP with a copy of the transcription regulatory sequence for ORF6 (TRS6) at the 5′ end of the EGFP sequence was inserted into plasmid pBAC-SD16^FL^-AM to produce plasmid pBAC-SD16^FL^-TRS6-EGFP.

Prior to introducing the full-length viral cDNA into the pBeloBAC11, pBAC-SD16-5′-3′ was assembled similarly as previously described by Almazan et al. and St-Jean et al. [46,47], using the pBeloBAC11 plasmid as the backbone. This plasmid includes the first 758 nucleotides (nts) of the genome under the control of the CMV promoter, and the last 2014 nts of the viral RNA, followed by a 32-bp synthetic poly (A) tail, the hepatitis delta virus ribozyme (HDV), and the bovine growth hormone termination and polyadenylation sequences (BGH) to lead an accurate 3′ end (Figure 1a). In addition, a multicloning site containing the restriction sites *Kas* I, *Pml* I, *Bmt* I, *Nsi* I, *Asc* I, and *Bsu36* I was introduced between the viral sequences and used to construct the full-length viral cDNA.

HP-PRRSV/SD16 was propagated in Marc-145 cells and total RNA was isolated from the infected cells using TRIZOL reagent (Invitrogen, Carlsbad, CA, USA). The entire viral genome of HP-PRRSV/SD16 except for the 5′ and 3′ ends was amplified by RT-PCR using Phusion® High-Fidelity PCR Master Mix (NEB, Ipswich, MA, USA) in five distinct overlapping regions (named F1 to F5) (Figure 1a). Each amplicon was inserted into the pEASY^™^-blunt simple cloning vector and after sequencing, introduced into the pBAC-SD16-5′-3′ vector to generate the pBAC-SD16^FL^ (Figure 1a). The unique restriction sites inserted into pBAC-SD16^FL^ between nt 15 170 and 15 171 of the HP-PRRSV/SD16 genome cDNA sequence were used for the cloning of EGFP under the control of the TRS6. To this end, fragment Fa (ending at the N gene with *AsiS* I and *Mlu* I sites introduced at the 3′ end) and Fm (starting from the 3′-UTR with *AsiS* I and *Mlu* I sites introduced at the 5′ end) were amplified from pBAC-SD16^FL^ and ligated together to generate fragment Fam, which was used to replace the fragment used in the original construction of pBAC-SD16^FL^ to generate plasmid pBAC-SD16^FL^-AM (Figure 1b). The sequence analysis revealed that two unique restriction sites (*AsiS* I and *Mlu* I) were correctly inserted into pBAC-SD16^FL^ between nt 15 170 and 15 171 of the HP-PRRSV/SD16 genome.

The EGFP gene was amplified from the pEGFP-N1 Vector (Clontech, Mountain View, CA, USA) using Phusion® High-Fidelity PCR Master Mix (NEB, Ipswich, MA, USA) with primers 5′-GCGATCGC**TGATGGTTCCGCGGCAACCCCTTTAACCAGAGTTTCAGCGGAACA**ATGGTGAGCAAGGGCGAGG -3′ (the *AsiS* I site is underlined), containing a copy of the TRS6 sequence (in bold), and 5′- CGACGCGTCGTTACTTGTACAGCTCGTCCA -3′ (the *Mlu* I site is underlined). The amplified product was inserted into the pEASY™-blunt simple cloning vector to generate plasmids pEASY-TRS6-EGFP, and after sequencing, cloned into *AsiS* I / *Mlu* I-cut pBAC-SD16^FL^-AM to generate plasmid pBAC-SD16^FL^-TRS6-EGFP (Figure 1c). All primer sequences used in this study are available from the corresponding author upon request.

### Transfection and rescue of recombinant viruses

To rescue the recombinant HP-PRRSV/SD16 and HP-PRRSV/SD16/TRS6-EGFP, 80% confluent Marc-145 cells cultured in 6-well plates were transfected with the plasmids pBAC-SD16^FL^ and pBAC-SD16^FL^-TRS6-EGFP using Attractene Transfection Reagent (Qiagen, Valencia, CA, USA) according to the manufacturer’s instructions. After 4–5 days of incubation at 37 °C, the cells and supernatants were collected and freeze-thawed for three times and the supernatants were then used to infect Marc-145 cells to propagate the rescued virus. The complete genomic sequences of the rescued viruses were confirmed by sequencing. The rescued viruses of HP-PRRSV/SD16 and HP-PRRSV/SD16/TRS6-EGFP were named rHP-PRRSV/SD16 and rHP-PRRSV/SD16/TRS6-EGFP, respectively.

### Propagation of recombinant viruses

Because rHP-PRRSV/SD16/TRS6-EGFP is much easier for evaluating the reverse genetic system than that for rHP-PRRSV/SD16, rHP-PRRSV/SD16/TRS6-EGFP was used in the following experiments. To determine whether the rescue procedure or exogenous gene insertion affected the replication of the recombinant viruses, the growth curves of rHP-PRRSV/SD16/TRS6-EGFP and parental virus were determined by serially passaging in Marc-145 with a multiplicity of infection (MOI) of 0.01. After 1 h of virus adsorption, the cells were washed three times with PBS and incubated in DMEM with 3% FBS at 37 °C in 5% CO2 incubator. The supernatants were collected at various time points and viral titres were determined using the Reed-Muench method.

### Immunofluorescence assays

Marc-145 cells grown on glass cover slips in a 24-well plate were infected with HP-PRRSV/SD16 or rHP-PRRSV/SD16/TRS6-EGFP at a MOI of 0.01 for 2 days. Cells were then fixed with 3% paraformaldehyde. A monoclonal antibody (6D10) to PRRSV N protein was used as the primary antibody and AlexaFluor 568-labelled anti-mouse IgG (Invitrogen, Carlsbad, CA, USA) was used as the secondary antibody. In addition, the cells were stained with 4-6-diamidino-2-phenylindole (DAPI) for 5 min at room temperature. Images were taken by sequential scanning at each wavelength on a Leica confocal microscope. Mock-infected cells were used as controls.

### Western blot

Marc-145 cells were infected with HP-PRRSV/SD16 or rHP-PRRSV/SD16/TRS6-EGFP at a MOI of 0.01 and cultured until 60% of cells showed the cytopathic effect (CPE). The infected cells were lyzed and the cellular proteins were separated by SDS-PAGE and blotted on a polyvinylidene difluoride (PVDF) membrane. The membrane was incubated with mouse anti-GFP monoclonal antibody, anti-β-Tubulin antibody (Abcam, Cambridge, MA, USA) and 6D10, as the primary antibodies, and then with peroxidase-conjugated goat anti-mouse IgG (Jackson, West Grove, PA, USA) as the secondary antibody. Immunostained proteins were visualized using ECL Western Blotting Substrate (Pierce, Rockford IL, CA, USA). Cellular proteins from mock-infected Marc-145 cells were used as controls.

### Quantification of EGFP fluorescence

To determine whether repeated passages of rHP-PRRSV/SD16/TRS6-EGFP in Marc-145 cells affected replication and expression, the recombinant virus was propagated in Marc-145 cells for 10 passages and assayed for total (cell-associated and released into culture media) virus titres and for EGFP expression using GFP Quantification Kit (BioVision, Mountain View, CA, USA), according to the manufacturer’s instructions. Briefly, Marc-145 cells grown in 6-well plates were infected with rHP-PRRSV/SD16/TRS6-EGFP at a MOI of 0.01. A total of 10^6^ cultured cells were freeze-thawed for three times when the cells showed 100% CPE and homogenized with 0.25 mL of assay buffer, incubated on ice for 10 min and centrifuged for 5 min at 15 000 × *g*. The supernatants and the medium collected from the infected cells were stored at -20 °C. A total of 100 μL samples in assay buffer was transferred to wells of a 96-well white plate (NUNC, Lowell, MA, USA). The EGFP fluorescence of each well was read on a Microplate Fluorescence Reader (BioTek, Winooski, VT, USA) at Ex/Em = 488/507 nm. The quantity of EGFP was determined by comparing its fluorescence with that of the GFP standard. Mock-infected cells were used as controls.

### In vivo imaging of PRRSV-EGFP expression

Marc-145 cells were infected with rHP-PRRSV/SD16/TRS6-EGFP at a MOI of 0.01 in a 35 mm cell culture dish. After 1 h post infection (pi), the medium was removed and replaced with 2 mL of pre-warmed DMEM containing 3% fetal calf serum. The dish was placed in the 37 °C observation chamber containing 5% CO_2_ (Leica CTR-Controller 3700, Wetzlar, Germany). EGFP fluorescence and phase contrast images were captured every 15 min for a period of 3 days by a live cell station (Leica AF6000, Wetzlar, Germany). Subsequently, images were processed into a movie of six frames s-1 using QuickTime Pro.

### Virus neutralisation assay

Marc-145 cells (2 × 10^2^/well) were cultured in 96-well plates. Swine antiserum to HP-PRRSV/SD16 and negative serum were inactivated by heating at 56 °C for 30 min before testing. The inactivated serum was serially two-fold diluted and 80 μL of each diluted serum was mixed with equal volume of rHP-PRRSV/SD16/TRS6-EGFP or wt HP-PRRSV/SD16 (100 TCID_50_/well) and then incubated at 37 °C for 1 h. The mixture of virus and serum was added to each of the wells plated with the monolayered Marc-145 cells and incubated at 37 °C for 1 h. The cells were washed twice with DMEM, and then cultured in DMEM containing 3% FBS at 37 °C, 5% CO_2_ for 48 h. The EGFP fluorescence and CPE were imaged using a fluorescence microscope (Leica, Germany).

### Applicability of EGFP-tagged HP-PRRSV to screen antiviral compound

The effect of antiviral compound CsA on HP-PRRSV replication was investigated. Marc-145 cells were infected with rHP-PRRSV/SD16/TRS6-EGFP (0.1 MOI) for 1 h. The plates were washed with DMEM and the various concentrations of CsA (0.25–64 μM) were added. After 36 h pi, Marc-145 cells were fixed and examined under the fluorescent microscope. The effect of CsA on cell viability was determined at 36 h after treatment with CsA using a CellTiter 96 A Q_ueous_ nonradioactive cell proliferation assay (Promega) according to the manufacturer’s instructions. To determine the effect of CsA treatment on the production of infectious virus progeny, Marc-145 cells were infected with rHP-PRRSV/SD16/TRS6-EGFP or HP-PRRSV/SD16 at 0.1 MOI and treated with CsA at various concentrations (2–8 μM) for 24 h. The virus was titered from the supernatants using the Reed-Muench method. To verify the inhibition of EGFP-tagged HP-PRRSV replication by CsA, Marc-145 cells were infected with rHP-PRRSV/SD16/TRS6-EGFP at 0.01MOI, and then treated with CsA at 2 to 16 μM for 1 h. At 36 h pi, cells were lysed and viral proteins were detected by Western blot analysis. Cellular proteins from mock-infected Marc-145 cells were used as controls.

## Results

### Rescue of rHP-PRRSV/SD16 and EGFP expression of rHP-PRRSV/SD16/TRS6-EGFP

The recombinant rHP-PRRSV/SD16 and rHP-PRRSV/SD16/TRS6-EGFP viruses were rescued successfully in Marc-145 cells and rHP-PRRSV/SD16/EGFP was used in the following experiments since it can be used for evaluating or optimizing the reverse genetics system. The recombinant rHP-PRRSV/SD16/TRS6-EGFP was recovered in 60–80% of Marc-145 cells transfected using the optimal ratios of plasmid and transfection reagent.

**Figure 2:**
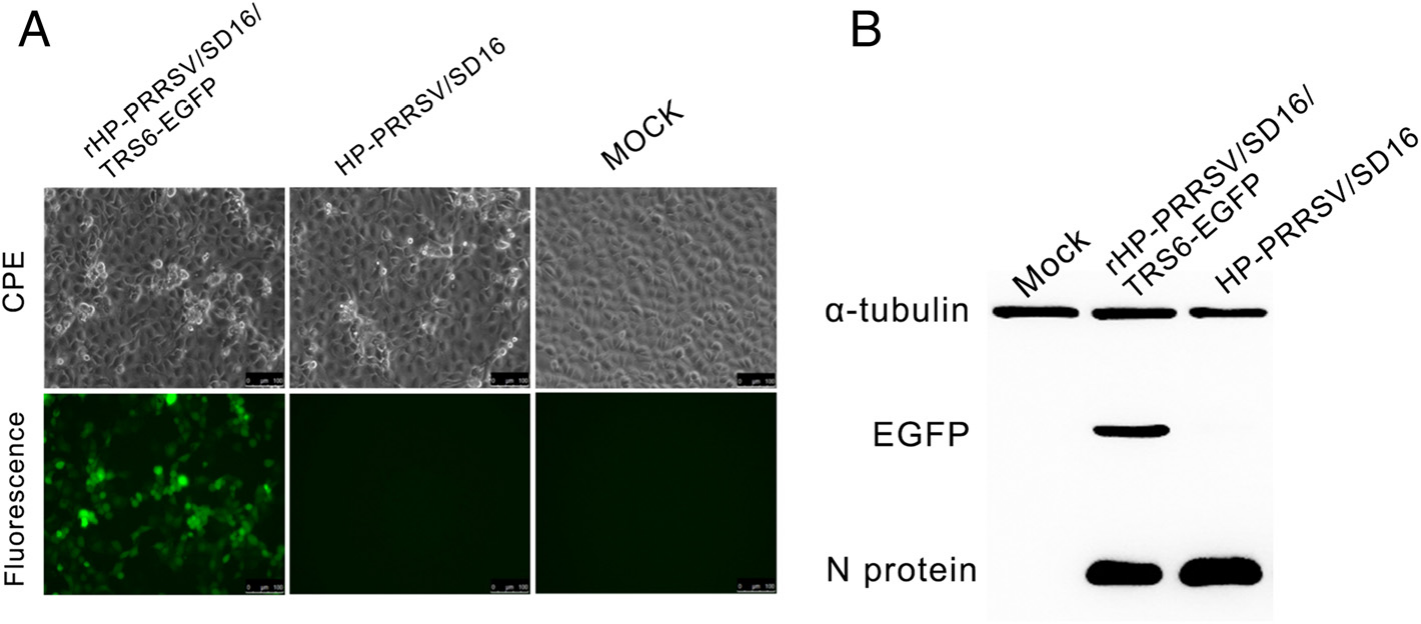
**Characterisation of recombinant rHP-PRRSV/SD16/TRS6-EGFP containing an encoding EGFP as a separate transcription unit. A**. Marc-145 cells infected with rHP-PRRSV/SD16/TRS6-EGFP and HP-PRRSV/SD16 were observed for CPE and fluorescence detection, respectively. Living cells were analysed by phase contrast and fluorescence microscopy. **B**. Western blot analysis of virus N protein and EGFP levels in Marc-145 cells infected with wild-type HP-PRRSV/SD16 or recombinant rHP-PRRSV/SD16/TRS6-EGFP.

Marc-145 cells transfected with pBAC-SD16^FL^-TRS6-EGFP showed a strong EGFP signal and infected cells were easily observed by live cell imaging (Figure 2a and Additional file 1). From the initial observations of the infected cells, it appears that EGFP was present in cells in early stages of infection when the CPE was not apparent. This clearly shows that only Marc-145 cells infected with rHP-PRRSV/SD16/TRS6-EGFP showed the expected green fluorescence (Figure 2a). To confirm that EGFP was expressed in rHP-PRRSV/SD16/TRS6-EGFP-infected cells, EGFP and PRRSV N protein expression was analysed by Western blot using anti-GFP and 6D10 antibodies, respectively. As shown in Figure 2b, normal viral protein expression and EGFP expression were simultaneously detected in rHP-PRRSV/SD16/TRS6-EGFP infected cells. However, EGFP expression was not detected in mock- or HP-PRRSV/SD16-infected Marc-145 cells (Figure 2b).

### Replication properties of recombinant viruses and stability of EGFP

**Figure 3:**
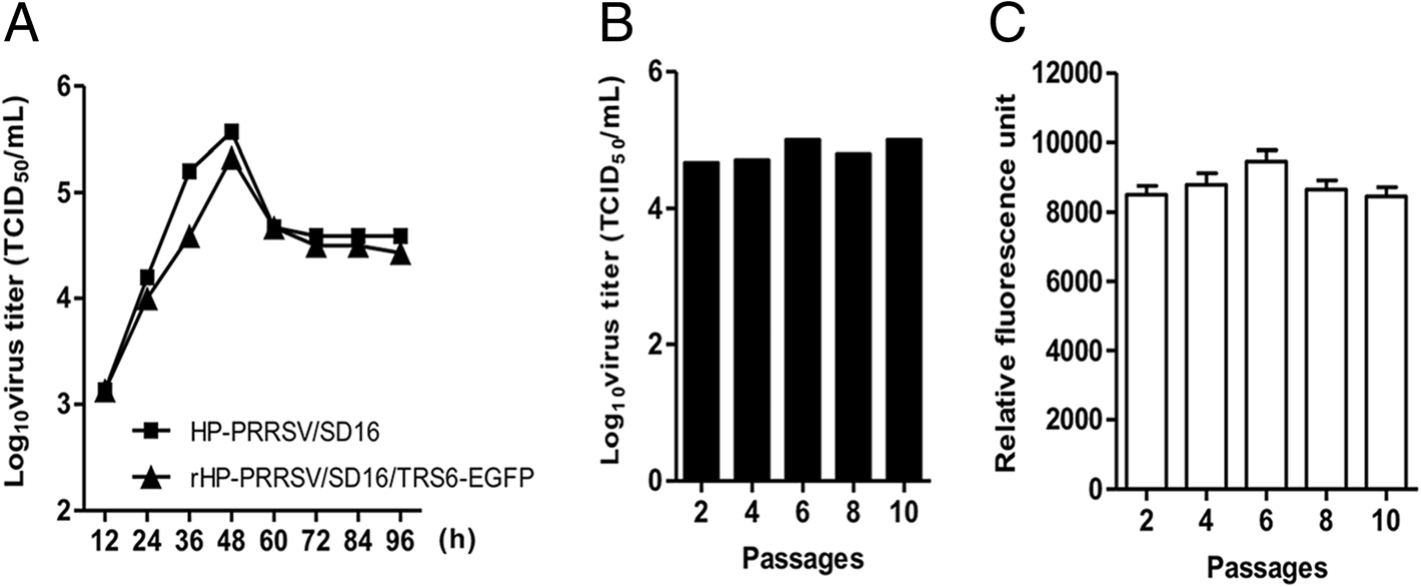
**Growth and stability of rHP-PRRSV/SD16/TRS6-EGFP in Marc-145 cells. A**. Marc-145 cells were infected with HP-PRRSV/SD16 or rHP-PRRSV/SD16/TRS6-EGFP and the amount of virus in the cultures was measured at various times from 12 to 96 h pi. **B**. rHP-PRRSV/SD16/TRS6-EGFP was passaged in Marc-145 cells every 3–4 days. The cells of each passage were freeze-thawed three times and used for virus titration. **C**. EGFP expression throughout virus passages in Marc-145 cells was analysed by quantitating relative fluorescence using a GFP Quantification Kit. Data were expressed as the mean ± standard deviation (SD).

To determine whether the rescue procedure or exogenous gene insertion affected the replicate ability of the recombinant virus, growth curves of HP-PRRSV/SD16 and rHP-PRRSV/SD16/TRS6-EGFP in Marc-145 cells were determined. The growth characteristics of rHP-PRRSV/SD16/TRS6-EGFP were evaluated in a time-course experiment and were compared with those of the parental virus. rHP-PRRSV/SD16/TRS6-EGFP growth rate and maximum titre were similar to those of the parental virus (Figure 3a). To determine whether repeated passage of rHP-PRRSV/SD16/TRS6-EGFP in Marc-145 cells affected replication and expression as well as the stability of the EGFP insertion, rHP-PRRSV/SD16/TRS6-EGFP was propagated and assessed by monitoring EGFP expression in Marc-145 cells for 10 passages. The samples of culture medium plus cells collected every second passage were assayed for total (cell-associated and released into culture media) virus titre and for EGFP expression quantification detection. The results show that the virus titre (Figure 3b) and EGFP expression (Figure 3c) from different passages was kept almost constant. To determine whether the EGFP gene was stable and no expected mutations or deletions were incorporated into the genome, intracellular RNA was isolated after infection with viruses from passages 5 and 10 (P5 and P10, respectively) and subjected to RT-PCR and direct sequence analysis. The results demonstrate that the intact EGFP gene was detected in both P5 and P10 samples, indicating that at least during propagation in cell culture rHP-PRRSV/SD16/TRS6-EGFP was stable and that its growth kinetics were similar to those of the parental virus.

### Cell to cell spread of rHP-PRRSV/SD16/TRS6-EGFP

The spread of rHP-PRRSV/SD16/TRS6-EGFP in Marc-145 cells was analysed by live-cell imaging to test whether the EGFP-tagged viruses are useful in real-time imaging. Marc-145 cells were infected with rHP-PRRSV/SD16/TRS6-EGFP at a MOI of 0.1 and the cell monolayer was analysed by collecting EGFP fluorescence and phase contrast photomicrographs. Photomicrographs of 10 different positions were captured every 15 min from 1 h pi for a period of 3 days (Additional file 1). Individual infected cells expressing EGFP were observed at about 10 h pi and EGFP expression in neighbouring cells was detected between 10 and 11 h later with a mean value of 10.5 h, which correlates with the PRRSV replication cycle of approximately 10 h [24]. Moreover, EGFP expression in surrounding cells was observed as late as 24 h pi in some infectious positions and EGFP fluorescence was distributed evenly on the monolayer in the late infection stage. In addition, observation of infected cells shows that EGFP was initially present in cells without CPE. Therefore, detection of fluorescence could be an early indicator of PRRSV infection in the cells. On one hand, single infected cells were observed in early infection, and then the infection spread to cells in close proximity to the initially infected cell (Additional file 1). On the other hand, virus spread could be promoted by viral replication in actively dividing cells (Additional file 1). Overall, these data suggest that PRRSV spread occurred by direct cell to cell contact rather than by release of infectious viral particles to the medium.

### Neutralisation of the recombinant virus

**Figure 4:**
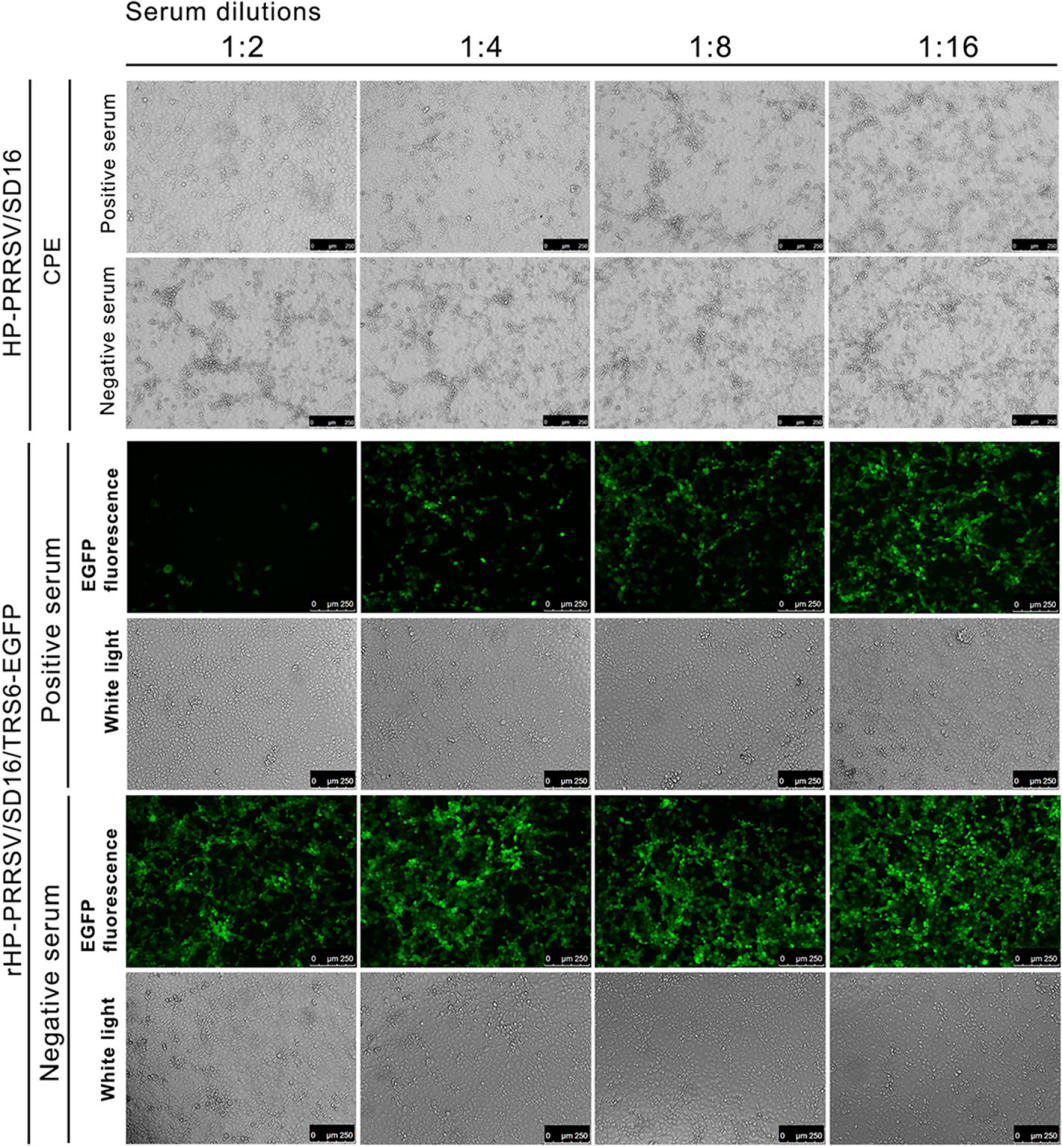
**Neutralisation of rHP-PRRSV/SD16/TRS6-EGFP infection.** The rHP-PRRSV/SD16/TRS6-EGFP and HP-PRRSV/SD16 viruses were incubated with serial dilutions of either a HP-PRRSV/SD16 antiserum or a negative serum, and then used to infect Marc-145 cells. The EGFP fluorescence and CPE were analysed at 48 h pi using a Leica confocal microscope at a magnification of × 250. Each serum dilution was tested in triplicate.

Antiserum from a pig infected with HP-PRRSV/SD16 was used to neutralise rHP-PRRSV/SD16/TRS6-EGFP and wt HP-PRRSV/SD16 infections of Marc-145 cells at 48 h pi. As shown in Figure 4, the antiserum diluted from 1:2 to 1:8 neutralised both rHP-PRRSV/SD16/TRS6-EGFP and wt HP-PRRSV/SD16 virus infection of Marc-145 cells as shown by EGFP fluorescence and CPE, whereas the negative serum did not neutralise the virus infection. These data indicate that rHP-PRRSV/SD16/TRS6-EGFP virus is a useful tool for screening neutralising antibodies.

### CsA inhibited the replication of EGFP-tagged HP-PRRSV

**Figure 5:**
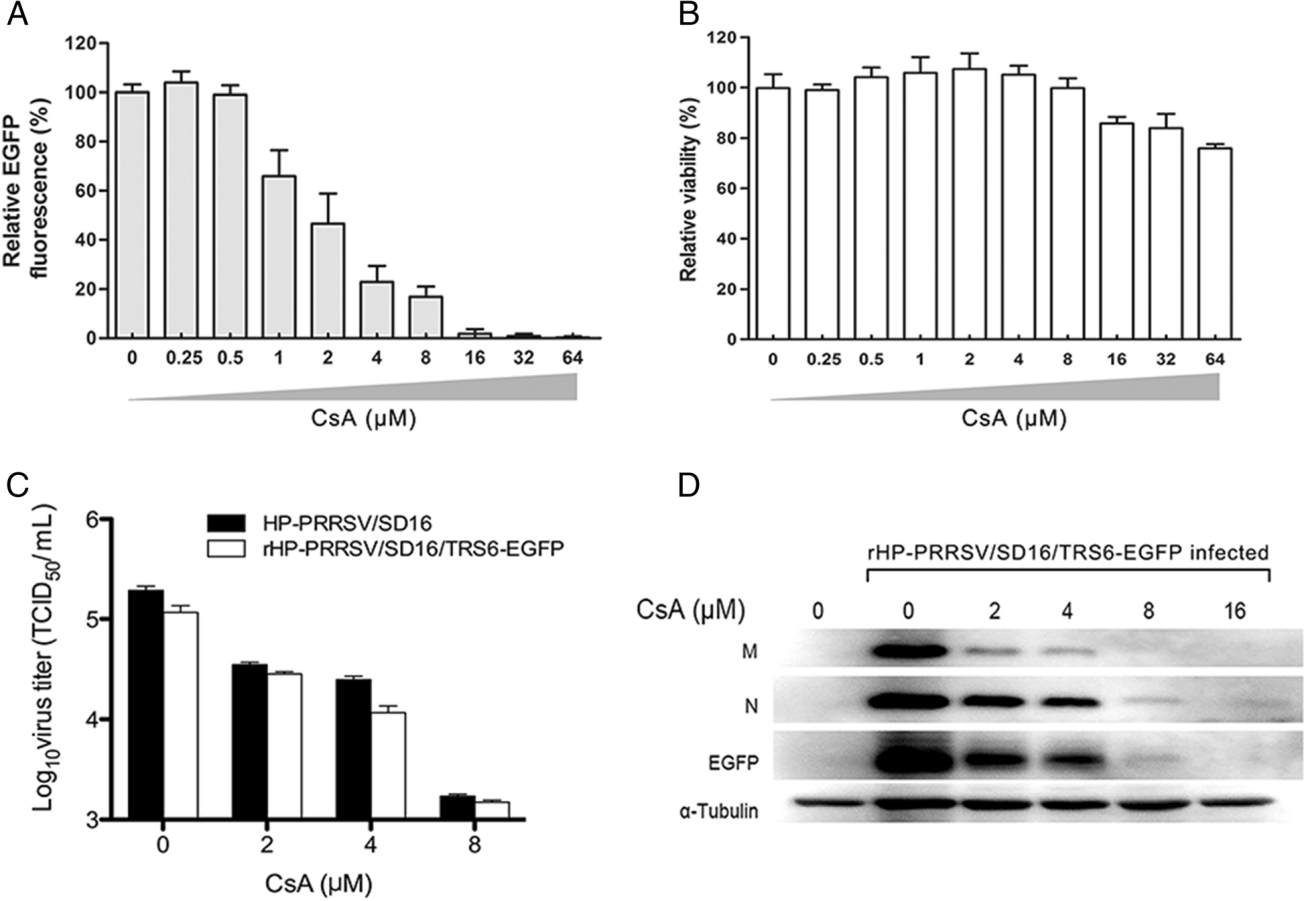
**CsA inhibits EGFP-tagged HP-PRRSV replication. A**. CsA at various concentrations inhibited rHP-PRRSV/SD16/TRS6-EGFP infection of Marc-145 cells as shown by the relative EGFP fluorescence expression normalised to the EGFP signal in solvent-treated control cells (100%). **B**. The effect of CsA at various concentrations on cell viability compared to the viability of untreated control cells (100%) was determined using a CellTiter 96 A Q_ueous_ nonradioactive cell proliferation assay at 36 h after treatment with CsA. **C**. CsA at various concentrations inhibited rHP-PRRSV/SD16/TRS6-EGFP and wt HP-PRRSV/SD16 infection of Marc-145 cells by determining the virus titers in the supernatants at 24 h pi. **D**. CsA at various concentrations inhibited the levels of rHP-PRRSV/SD16/TRS6-EGFP structural proteins N and M and EGFP protein by Western blot analysis.

The effect of CsA on HP-PRRSV replication was investigated using rHP-PRRSV/SD16/TRS6-EGFP and wt HP-PRRSV. As shown in Figure 5a, CsA inhibited HP-PRRSV/SD16/TRS6-EGFP infection of Marc-145 cells in a dose dependent manner as indicated by the EGFP signal. At the concentration of 16 μM, the EGFP signal was hardly detected (Figure 5a), and the replication of rHP-PRRSV/SD16/TRS6-EGFP was completely blocked by CsA at 64 μM. CsA had only a slight effect on cell viability (Figure 5b). The production of rHP-PRRSV/SD16/TRS6-EGFP and wt HP-PRRSV infectious progeny were greatly reduced by CsA at the concentrations of 2 μM to 8 μM in a dose dependent manner (Figure 5c). The effect of CsA on the production of the viral proteins was evaluated by the Western blot. As shown in Figure 5d, the levels of viral structural proteins M and N and EGFP proteins were markedly reduced by CsA in a dose dependent manner.

## Discussion

Here, we report the rescue of a recombinant HP-PRRSV carrying an EGFP reporter gene as a separate transcription unit between the N gene and 3′-UTR. For the construction of HP-PRRSV as an expression vector, we first selected the sites of insertion. American types of PRRSV have only two non-overlapping regions located between ORF1b and ORF2a, as well as between ORF4 and ORF5 of the genome. Since these intergenic regions are reduced in size (about 11 to 220 nts), the insertion of an extra cassette could alter the expression of the ORF downstream from the insertion site due to the proximity to the TRS of the next gene [48]. Except for the intergenic regions, the extra cassette can also be inserted just before the ORF1, or after the ORF7 regions since there were no overlapping genes. Insertions before ORF1 may block the early translation of pp1a and pp1ab, which are responsible for the subsequent replication and formation of subgenomic mRNA. As a result, the site between N gene and 3′-UTR was chosen for the insertion. Secondly, the TRS6 was chosen because it had the shortest distance (24-bp) between the body TRS and downstream ORF. Taken together, this position was selected for the insertion of a copy of the TRS6 in order to drive transcription of EGFP as a separate transcription unit.

In previous studies, recombinant PRRSV including a foreign HA tag fused to ORF7 started to lose the introduced epitope at passage 2 and continued to lose with the increasing number of passages [43]. In a recombinant PRRSV expressing GFP gene fused to the nsp2 gene, the heterologous insertion was lost after 7 serial passages due to a N-terminal deletion of GFP from aa 1 to 159 [44]. Similarly, the foreign EGFP gene was lost after 5 serial passages due to a single aa mutation [45]. However, a recombinant PRRSV expressing GFP gene with an extra TRS-ORF cassette inserted between ORF1ab and ORF2 was stable even after 37 serial passages using TRS2 to drive transcription of the GFP gene and inserting an extra synthetic TRS6 to drive transcription of the ORF 2a and 2b genes [22]. Taken together, these data show that PRRSV can tolerate the addition of a foreign gene at specific positions, although the insertion of extra non-viral genetic material may lead to the reduction of the level of the viral replication or result in variants that no longer encode a functional foreign gene. In an attempt to circumvent these disadvantages and to reduce to a minimum the modification of the PRRSV genome, we cloned the heterologous gene between N gene and 3′ UTR using a BAC approach to recover recombinant HP-PPRSV [46,47,49-52]. The EGFP was introduced into the HP-PRRSV/SD16 genome with limited modification of the genome to create a marked recombinant PPRSV, rHP-PRRSV/SD16/TRS6-EGFP, which stably expressed EGFP as a separate transcription unit. Importantly, rHP-PRRSV/SD16/TRS6-EGFP shows similar patterns in growth rate and maximum titre in comparison with the parental virus.

Our results show that Marc-145 cells transfected with pBAC-SD16^FL^-TRS6-EGFP produced both green fluorescent cells and viral progeny. Insertion of EGFP as a separate transcription unit did not affect replication of the recombinant virus in the infected cells. Furthermore, the fact that EGFP fluorescence in EGFP recombinant viruses was expressed stable and at higher levels in different passages, suggests that the cDNA infectious clone represents a useful tool for construction of potential recombinant vaccine candidates, in which the EGFP ORF can be replaced by sequences encoding immunogenic antigens of other viruses. Overall, the virus recovered from the infectious cDNA clone exhibited the same genotypic and phenotypic properties as the parental virus.

The infection of cells with rHP-PRRSV/SD16/EGFP can be used as an excellent model to study the release of infectious PRRSV and virion formation in infected cells. Early observation of infected cells showed that EGFP was expressed in cells with no apparent CPE. This could be due to high expression levels associated with genes located close to the genome end. In addition, EGFP may passively spread into adjacent cells through cell-to-cell-contact from the earlier infected cells. Therefore, the detection of fluorescence could be more sensitive than PRRSV antigen detection. Finally, PRRSV replication was observed in actively dividing Marc-145 cells, indicating that PRRSV could infect quiescent and dividing cells. These results clearly show that EGFP fluorescence can be an early indicator of the infection in cells and a useful tool to show the processes of the rapid spread of PRRSV from cell to cell. In addition, the rHP-PRRSV/SD16/TRS6-EGFP was found to be an excellent tool for screening neutralising antibodies.

Previously, the drug CsA was demonstrated to inhibit the replication of a variety of RNA viruses using different pathways including herpes simplex virus, human immunodeficiency virus type 1, hepatitis C virus and influenza virus [53-56]. In the present study, we demonstrate that CsA inhibited the HP-PRRSV replication in Marc-145 cells in a dose dependent manner (Figure 5). These results were in accordance with a previous study showing that CsA inhibits arterivirus replication by interfering with viral RNA synthesis [57].

In conclusion, we report the rescue of a recombinant PRRSV carrying an EGFP reporter gene as a separate transcription unit. The PRRSV reverse genetics system used is highly flexible and may facilitate the engineering of recombinant multivalent vaccines by replacing the EGFP ORF sequences encoding immunogenic antigens from another virus. The generation of an EGFP- expressing HP-PRRSV could be a useful tool not only to monitor virus spread and screen for antiviral compounds and neutralising antibodies, but also for fundamental research on the biology of the virus.

## Competing interests

The authors declare that they have no competing interests.

## Authors’ contributions

CBW, BCH, and EMZ carried out all the experiments and drafted the manuscript. CL and LD cloned and sequenced PRRSV genomic RNA. NK and YPM constructed the plasmid and performed Western blot analysis. CZ and JMG carried out the live-cell imaging microscopy. QL performed the cell culture and quantification analysis of EGFP fluorescence. WXP, SQX, YM, QZ, and YNS carried out the CsA inhibition analysis. FA and LE engineered a BAC backbone to clone PRRSV cDNA and interpreted the data. All authors critically reviewed the manuscript and provided final approval.

## Supplementary Material

Additional file 1 **Live imaging of rHP-PRRSV/SD16/TRS6-EGFP observed by in vivo live-cell microscopy.** Marc-145 cells were infected at an MOI of 0.01 in a 35 mm cell culture dish with rHP-PRRSV/SD16/TRS6-EGFP. EGFP fluorescence signals were acquired every 15 min for a period of 3 days using a live cell station, as described in the Materials and methods. Images were processed into a movie of six frames s-1 using QuickTime Pro. Scale bar, 100 μm. Click here for file
